# Meta-analysis of Plasmodium falciparum *var* Signatures Contributing to Severe Malaria in African Children and Indian Adults

**DOI:** 10.1128/mBio.00217-19

**Published:** 2019-04-30

**Authors:** Fergal Duffy, Maria Bernabeu, Prasad H. Babar, Anne Kessler, Christian W. Wang, Marina Vaz, Laura Chery, Wilson L. Mandala, Stephen J. Rogerson, Terrie E. Taylor, Karl B. Seydel, Thomas Lavstsen, Edwin Gomes, Kami Kim, John Lusingu, Pradipsinh K. Rathod, John D. Aitchison, Joseph D. Smith

**Affiliations:** a Seattle Children's Research Institute, Seattle, Washington, USA; b Goa Medical College & Hospital, Bambolim, Goa, India; c Departments of Chemistry and Global Health, University of Washington, Seattle, Washington, USA; d Albert Einstein College of Medicine, Bronx, New York, USA; e Centre for Medical Parasitology, Department of Immunology and Microbiology, University of Copenhagen, Copenhagen, Denmark; f Department of Infectious Diseases, Copenhagen University Hospital (Rigshospitalet), Copenhagen, Denmark; g Malawi-Liverpool Wellcome Trust Clinical Research Programme, Blantyre, Malawi; h College of Medicine, Biomedical Department, University of Malawi, Blantyre, Malawi; i Academy of Medical Sciences, Malawi University of Science and Technology, Thyolo, Malawi; j Department of Medicine at the Doherty Institute, The University of Melbourne, Melbourne, Australia; k Blantyre Malaria Project, University of Malawi College of Medicine, Blantyre, Malawi; l College of Osteopathic Medicine, Michigan State University, East Lansing, Michigan, USA; m Tanga Medical Research Centre, National Institute for Medical Research, Tanga, Tanzania; n Institute of Systems Biology, Seattle, Washington, USA; o Department of Global Health, University of Washington, Seattle, Washington, USA; NIAID/NIH

**Keywords:** cerebral malaria, machine learning, malaria, PfEMP1, Plasmodium falciparum, severe malaria, *var* gene

## Abstract

The clinical presentation of severe Plasmodium falciparum malaria differs between children and adults, but the mechanistic basis for this remains unclear. Contributing factors to disease severity include total parasite biomass and the diverse cytoadhesive properties mediated by the polymorphic *var* gene parasite ligand family displayed on infected erythrocytes. To explore these factors, we performed a multicohort analysis of the contribution of *var* expression and parasite biomass to severe malaria in two previously published pediatric cohorts in Tanzania and Malawi and an adult cohort in India. Machine learning analysis revealed independent and complementary roles for *var* adhesion types and parasite biomass in adult and pediatric severe malaria and showed that similar *var* profiles, including upregulation of group A and DC8 *var*, predict severe malaria in adults and children. Among adults, patients with multiorgan complications presented infections with significantly higher parasite biomass without significant differences in *var* adhesion types. Conversely, pediatric patients with specific complications showed distinct *var* signatures. Cerebral malaria patients showed broadly increased expression of *var* genes, in particular group A and DC8 *var*, while children with severe malaria anemia were classified based on high transcription of DC8 *var* only. This study represents the first large multisite meta-analysis of *var* expression, and it demonstrates the presence of common *var* profiles in severe malaria patients of different ages across distant geographical sites, as well as syndrome-specific disease signatures. The complex associations between parasite biomass, *var* adhesion type, and clinical presentation revealed here represent the most comprehensive picture so far of the relationship between cytoadhesion, parasite load, and clinical syndrome.

## Abstract (precis)

P. falciparum malaria can cause multiple disease complications that differ by patient age. Previous studies have attempted to address the roles of parasite adhesion and biomass in disease severity; however, these studies have been limited to single geographical sites, and there is limited understanding of how parasite adhesion and biomass interact to influence disease manifestations. In this meta-analysis, we compared parasite disease determinants in African children and Indian adults. This study demonstrates that parasite biomass and specific subsets of *var* genes are independently associated with detrimental outcomes in both childhood and adult malaria. We also explored how parasite *var* adhesion types and biomass play different roles in the development of specific severe malaria pathologies, including childhood cerebral malaria and multiorgan complications in adults. This work represents the largest study to date of the role of both *var* adhesion types and biomass in severe malaria.

## INTRODUCTION

Despite increased malaria control and elimination efforts, severe malaria (SM) from Plasmodium falciparum remains responsible for more than 400,000 deaths every year (1). In areas with high P. falciparum transmission, such as sub-Saharan Africa, SM primarily occurs in pediatric patients, as older children acquire protective immunity to the pathogenic effects of infection. In areas of low transmission, such as Asia and the Americas, SM occurs in both children and adults. This distinction is important, as malaria disease presentation varies between children and adults (2). Whereas cerebral malaria (CM) and metabolic acidosis are common to children and adults, severe malarial anemia is more common in children, and acute kidney injury, jaundice, and acute respiratory distress syndrome are most commonly seen in patients greater than 10 years old (2). The parasite and host factors that contribute to variability in malaria disease presentation remain poorly understood.

A key virulence determinant of P. falciparum is sequestration of infected erythrocytes (IEs) within the microvasculature (3). Extensive microvascular obstruction from sequestered IEs is believed to contribute to metabolic acidosis (4), while high levels of parasite sequestration in brain (5–7) and the placental intervillous space (8) are associated with organ-specific complications. Cytoadhesion of P. falciparum*-*IEs is mediated by the large and diverse P. falciparum erythrocyte membrane protein 1 (PfEMP1) family, encoded by *var* genes. PfEMP1 proteins are expressed in a clonally variant fashion on the surface of IEs (9–11). Each parasite genome contains ∼60 *var* genes that are classified into group A, B, or C based on chromosomal localization and the gene upstream region (12). Molecular insight into PfEMP1 function has been gained by sequence classification of the extracellular Duffy binding-like (DBLα/β/γ/δ/ε/ζ) and cysteine-rich interdomain region (CIDRα/β/γ/δ) adhesion domains (13, 14). CIDRα1 domains bind endothelial protein C receptor (EPCR) (15, 16), CIDRα2-6 domains bind to CD36 (17–19), and CIDRβ/γ/δ domains bind neither receptor (reviewed in reference 20). Additionally, PfEMP1 can simultaneously bind to other coreceptors, as some DBLβ1/3 (group A) and DBLβ5 (group B and C) domains mediate binding to ICAM-1 (21–25). PfEMP1 also includes sets of domains typically found tandemly arrayed in the same protein, termed domain cassettes (DCs). Several DCs encode conserved cytoadhesion traits, including binding to EPCR (DC8 and DC13) (16, 21, 23, 26, 27), ICAM-1 (DC4) (28), and PECAM-1 (DC5) (29). Antigenic switching of PfEMP1 proteins modifies P. falciparum-IE specificity for receptors on endothelial cells (cytoadhesion) or erythrocytes (termed rosetting [30]).

A challenge of studying the epidemiological associations of the *var* gene family and malaria disease is the immense diversity of *var* genes in the parasite population (31, 32). The development of primer sets targeting different *var* domain subtypes represented a breakthrough in malaria pathogenesis research (13, 33). This approach has shown that transcription of *var* genes encoding predicted EPCR binding activity is elevated in both pediatric (23, 33–36) and adult (37) SM patients. Moreover, deep sequencing of *var* amplicons has indicated that SM infections comprise a mixed population of parasites expressing different *var* genes (34, 38). Although these findings implicate specific PfEMP1 subsets in disease severity, these studies were insufficiently powered to assess whether expression of distinct PfEMP1 adhesion types are linked to different organ complications in children and adults. High parasite burden is also thought to play an important role in disease severity. Plasma levels of P. falciparum histidine-rich protein 2 (PfHRP2), a parasite protein released upon merozoite egress, is used as a blood surrogate marker for the combined amount of both circulating ring-stage and sequestered mature-stage P. falciparum-IEs. PfHRP2 levels are associated with severe malaria in both children and adults (39, 40), predict disease progression (41), and are increased in patients with specific complications, including cerebral malaria (42), and metabolic acidosis (43).

This study extends previous work (i) by applying an integrated multicohort analysis to better understand how both parasite burden and binding phenotype are associated with severe malaria in both adults and children and (ii) by examining how parasite burden and parasites expressing different *var* gene subsets influence disease severity and the spectrum of disease across study sites. To accomplish this, we performed a machine learning meta-analysis of combined *var* transcript profiles from two previously described African children cohorts (33, 34), along with an expanded Indian adult cohort based on our previously published work (37). This work represents the largest and broadest analysis of these parasite factors in severe malaria to date, thus providing unprecedented power to explore and compare the parasite factors that lead to severe disease.

## RESULTS

### Characteristics of the study cohorts.

The *var* gene transcript profiles (*var* profiles) for this work were obtained from two previously published studies of pediatric malaria in Tanzania (TZ) (33) and Malawi (BLZ) (34) and one adult malaria cohort in Goa, India (GMC). The cohorts included 90 (TZ), 68 (BLZ), and 55 (GMC) SM patients and 32 (TZ), 40 (BLZ), and 37 (GMC) uncomplicated malaria (UM) patients. The GMC cohort expanded our previously published (37) cohort with newly obtained samples, comprising both severe and uncomplicated malaria.

Previous work has shown that malaria disease presentation is influenced by both transmission intensity and patient age (2, 44). The recruited individuals in the three sites had different age profiles (see Table S1 in the supplemental material). Tanzanian UM and SM pediatric patients (median age, 2 years; range, 1 to 3 years) were generally younger than Malawian patients (median age, 4 to 4.5 years; range, 2 to 7 years) (Table S1). Goan adult UM and SM patients had median ages of 25 and 27 years, respectively (Table S1). UM and SM patients in all three cohorts did not show significant differences in circulating parasite density by blood smear. However, UM patients had significantly reduced parasite biomass compared to SM patients, as reflected by plasma PfHRP2 levels (Table S1). PfHRP2 levels were not measured in the TZ cohort. Patient mortality rates for SM were similar at all sites, ranging from 10% in TZ and BLZ patients to 13% in GMC patients (Table S1).

10.1128/mBio.00217-19.3 TABLE S1 Demographic and clinical characteristics of the study cohorts. Download Table S1, PDF file, 0.1 MB. Copyright © 2019 Duffy et al. 2019 Duffy et al. This content is distributed under the terms of the Creative Commons Attribution 4.0 International license.

### *var* transcript profiles and cross-site correlations.

**FIG 1:**
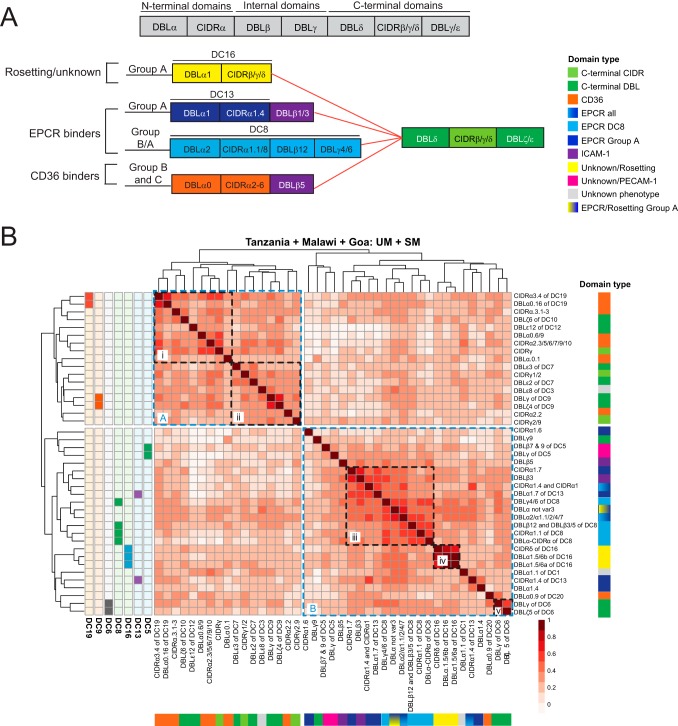
Correlation of *var* domain transcript profiles across the three study sites. (A) Schematic of PfEMP1 domain architecture illustrating the relationship between known binding phenotypes and different *var* groups. CIDRβ/γ/δ domains (yellow) have unknown binding properties, CIDRα1 domains (light and dark blue) bind EPCR, CIDRα2-6 domains (orange) bind CD36, and DBLβ1/3/5 domains (purple) bind ICAM-1. (B) Transcriptional profiling of *var* genes was performed with 41 different primer sets targeting different *var* domain subtypes. Shown is a heat map of correlations (Spearman’s rho) of transcript levels of different *var* domain subtypes across all samples (SM and UM) from all three sites. Domain transcript levels were hierarchically clustered, and known tandem domain arrangements (e.g., DC19) are indicated to the left of the heat map. The right and bottom legends use the color scheme described in Table S2 to indicate EPCR/CD36/ICAM-1/Rosetting/PECAM-1/unknown/C-terminal domains. Clusters A and B and subclusters i to v are highlighted in dashed boxes.

To compare the *var* adhesion types associated with malaria disease severity at the three sites, *var* transcript profiling was performed using a set of 41 PCR primer pairs designed to quantify transcripts of defined *var* domain subtypes (33), many of which have predicted binding phenotypes (33–35, 37) (Fig. 1A). The three cohorts analyzed in this meta-analysis were chosen as they employed the same primer panel enabling comparative analysis across sites. Table S2 in the supplemental material shows the primers used for these analyses, along with a color code based on predicted binding phenotypes of the targeted domain(s) used in all figures throughout this study. To examine how the primer sets performed across different parasite populations in Africa and India, correlations between transcript expression of individual *var* domain subtypes across every patient were calculated in all three sites and used to hierarchically cluster domains (Fig. 1B, left). Notably, the transcript expression of *var* domain subtypes grouped according to PfEMP1 domain architecture and *var* adhesion type. Tandemly arrayed domains (domain cassettes [DCs]) remain closely correlated at all three sites (i.e., DC5, DC6, DC9, DC16, and DC19), all but DC5 retain their close association across every site, and DC5 primers remain the most closely associated in Tanzania (Fig. 1B). For DC8, three of the four tandemly associated domains grouped together, and DC13 is the sole domain cassette whose two characteristic domains are not closely correlated with each other (Fig. 1B), probably reflecting the weaker association of these domain types (DBLγ4/6 [DC8] and DBLα1.7 [DC13]) with their domain cassettes (13). Clustering of DCs also holds true in each individual site, with the exception of DC5 (see Fig. S1 in the supplemental material). This analysis suggests that these protein architectural arrangements are maintained across diverse parasite populations in Africa and India, despite extensive gene recombination in the *var* gene family (45). This finding also suggests that tandem domain relationships may be under strong biological selection. Additionally, *var* transcripts clustered according to predicted *var* adhesion types (Fig. 1B). Cluster A was dominated by *var* genes encoding predicted CD36 binding domains (preferentially found in subcluster i) and C-terminal domains (preferentially found in subcluster ii). In contrast, cluster B contained *var* genes encoding predicted EPCR binding domains (subcluster iii) and *var* genes associated with unknown/rosetting function (subcluster iv). Exceptions in cluster B were subtypes relating to DBLα0.9 of DC20 and DC6 (subcluster v). Although DC6 domains can be found in all three *var* groups (13), its expression was more closely correlated to the DC8 and group A *var* genes across the three cohorts. This analysis suggests that transcription of specific *var* adhesion types is more closely correlated and that DCs are conserved across parasite populations of distant geographical sites.

10.1128/mBio.00217-19.1 FIG S1 Clustergrams of *var* domain subtypes related by expression correlation for each study site, similar to Fig. 1. Heat maps indicate *var* domain subtypes known to be part of defined domain cassettes. Download FIG S1, EPS file, 2.3 MB. Copyright © 2019 Duffy et al. 2019 Duffy et al. This content is distributed under the terms of the Creative Commons Attribution 4.0 International license.

10.1128/mBio.00217-19.4 TABLE S2 Broad phenotypic classification of domains targeted by individual *var* primers and associated color scheme. Download Table S2, PDF file, 0.1 MB. Copyright © 2019 Duffy et al. 2019 Duffy et al. This content is distributed under the terms of the Creative Commons Attribution 4.0 International license.

### Cross-site correlations in *var* transcript profiles.

**FIG 2:**
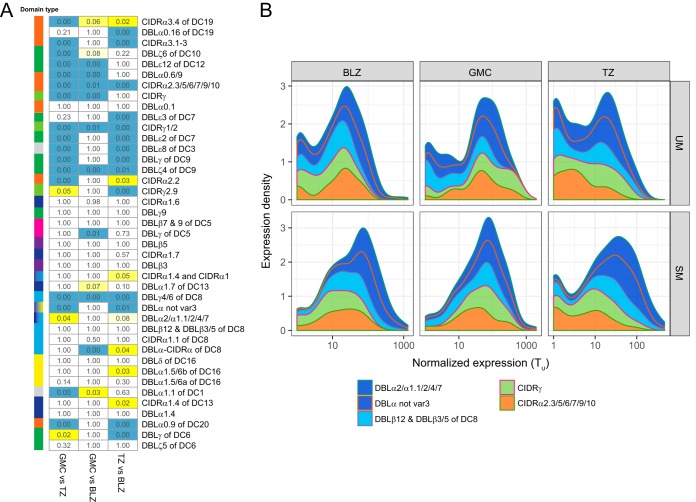
Correlations in *var* transcript levels across the three study sites. (A) Kolmogorov-Smirnov *P* values comparing *var* domain subtype distributions between each pair of sites. *P* values under 0.01 are highlighted in blue, and those under 0.05 are highlighted in yellow. Subtype color annotations and order are identical to those in Fig. 1B. (B) Transcription levels of the top five transcribed *var* domain subtype transcripts (median T_u_, >10) are stratified by site and severity (SM/UM).

To explore *var* transcript profiles across the three study sites and to assess the comparability of *var* profiles derived from each site, we compared *var* domain subtype expression distributions from each cohort. Overall, *var* genes encoding EPCR binding (both DC8 and group A *var*) were the highest expressed transcripts across the three sites (transcription units [T_u_] value = 10 to 1,000) (see Fig. S2 in the supplemental material). Differences in expression were tested by using the Kolmogorov-Smirnov test (Fig. 2A). While some domain subtypes presented similar patterns at all sites (*P* = 1.00), other domain subtypes showed site-specific differences in expression distributions. These differences are not solely explained by differences between patient age and ethnicity. For example, while adult GMC and pediatric BLZ patients shared 26 similarly distributed domain subtypes (*P* = 1.00), only 14 subtypes met this threshold when comparing pediatric TZ versus BLZ patients. These results were unexpected, as pediatric and adult severe malaria patients present different organ complications.

10.1128/mBio.00217-19.2 FIG S2 Transcript levels of the *var* domain subtypes targeted by the 41 primer sets vary according by study site and malaria status. Expression density plots for each *var* domain subtype were stratified by site. BLZ, Malawi; GMC, Goa; TZ, Tanzania. The *var* domain subtypes are shown in the same order as in Fig. 1B. Download FIG S2, EPS file, 8.1 MB. Copyright © 2019 Duffy et al. 2019 Duffy et al. This content is distributed under the terms of the Creative Commons Attribution 4.0 International license.

While EPCR-binding domains presented similar expression profiles across sites (*P* = 1.00), the main site-specific expression differences were observed for CD36-binding and C-terminal domains (Fig. 2A). Figure 2B shows the expression density distributions detected by primer pairs, stratified by study site and severity. We looked for differences in the transcript levels of the most abundantly transcribed *var* subtypes (median T_u_, >10) in the combined severe versus uncomplicated malaria cases. Five primer pairs were identified that passed this threshold: primer pair DBLα2/α1.1/2/4/7 targets all EPCR binders (DC8 and group A), primer pair DBLα not *var3* targets group A PfEMP1 (EPCR, rosetting, PECAM-1, unknown), primer pairs DBLβ12 and DBLβ3/5 mostly target DC8 (EPCR), and primer pairs CIDRγ and CIDRα2.3/5/6/7/9/10 target CD36-binding *var* genes. Notably, the rank order of the top 5 transcribed domains was the same in both SM and UM patients across the three sites, but the *var* transcript levels tended to be higher in SM patients (Fig. 2B). Two of the three EPCR-binding domains showed significantly higher expression in SM for all sites (DBLα not *var3*, BLZ, *P* = 8.8 × 10^−6^; GMC, *P* = 0.02; TZ, *P* = 2.5 × 10^−8^; DBLβ12 and DBLβ3/5 of DC8 BLZ, *P* = 1.6 × 10^−4^; GMC, *P* = 0.0004; TZ, *P* = 0.002), while neither of the CD36 high expressers showed any significant difference. In all three sites, UM patients showed a bimodal distribution of the DC8 and group A *var* transcripts, including two peaks representing low-expression and high-expression individuals (Fig. 2B). However, the low expressers’ peak is absent in SM cases from BLZ and GMC and highly diminished in TZ (Fig. 2B). Thus, even though individual domain subtype expression varies in a site-specific manner, marked increases in abundances of some PfEMP1 domains are a feature of SM across all study sites.

### Common *var* profiles predict severe malaria in both adults and children.

In order to more precisely evaluate the contribution of *var* transcript profiles to malaria severity, a machine learning approach was applied. Random forest (RF) models were trained on *var* profiles to classify samples as severe or uncomplicated malaria. This was performed for all sites individually and for Tanzania and Malawi together to derive a model representing all childhood cases.

**FIG 3:**
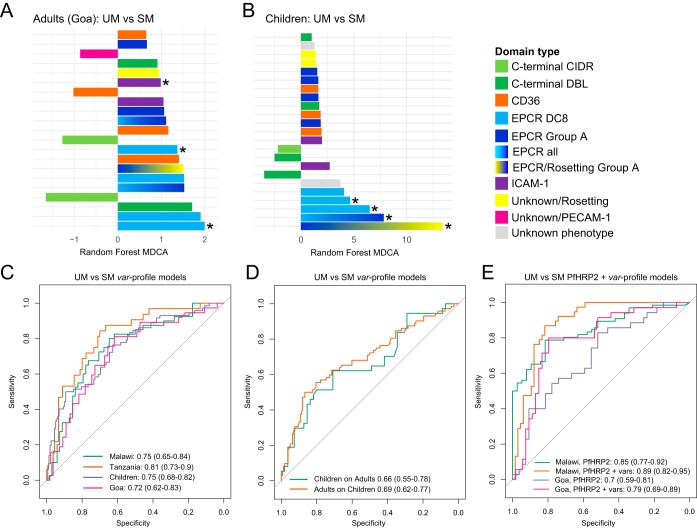
Parasite load and *var* adhesion types independently predict severe malaria in adults and children. (A and B) Bar plots showing *var* domain subtype model importance (measured as MDCA) for child and adult UM versus SM models. Each bar represents a single domain subtype targeted by one of the 41 primer sets used to generate the RF model, colored by predicted binding phenotype or position in the protein (see also Table S2 and Table S3). Positive MDCA indicates higher expression of a specific domain subtype in SM and vice versa. Bars with asterisks indicate that these domain subtypes showed significant differences in expression (false-discovery rate [FDR] of ≤0.2) using the mProbes algorithm. (C) ROC curves showing out-of-bag predictions of RF models classifying samples as SM or UM. One model was trained per site, along with a total childhood model combining Malawi and Tanzania. Performance is shown as area under the ROC curve, with 95% confidence intervals in parentheses. (D) ROC curves showing blind predictive performance of the child model predicting adult severe malaria, and the adult model predicting child severe malaria. (E) ROC curves showing predictive performance of serum PfHRP2 levels alone to classify severe malaria and PfHRP2 combined with the *var* profile RF models.

The importance of each adhesion domain to the adult and child RF models is shown in Fig. 3A and B. Importance was measured as the mean decrease in classifier accuracy (MDCA) when the primer is excluded from the model while resampling during model training. As previously reported, in both adults (37) and children (23, 33–36), SM is associated with increased transcription of domains predicted to bind EPCR. Despite comprising only 10% of the *var* repertoire (13), more than 50% of the top 10 predictive features in both models belong to PfEMP1-EPCR binders (Fig. 3A and B). Both the group A and DC8 subsets of EPCR binding *var* genes were predictive of pediatric SM (group A: DBLα not *var3*; DC8: DBLβ12 and DBLβ3/5, DBLα-CIDRα, and CIDRα1.1; and DC8-group A: DBLα2/α1.1/2/4/7). Although we previously reported that only DC8-EPCR binders were predictive of adult SM (37), both DC8 domains (DBLγ4/6, DBLβ12 and DBLβ3/5, DBLα-CIDRα, CIDRα1.1, and DBLα2/α1.1/2/4/7 [see Table S3 in the supplemental material]) and group A-EPCR binders (DBLα not *var3* and DBLα2/α1.1/2/4/7 [Table S3]) contributed to the adult SM model in the expanded data set. Additionally, *var* genes containing the DC19 subset of CD36 binding domains (children, CIDRα3.4; adults, DBLα0.16 [Table S3]) ranked among the top 10 predictive features in both the adult and children SM models. As well, high expression of *var* genes containing the C-terminal DC6 (DBLγ [Table S3]), which ranked as the top *var* feature in our previously published adult SM model (37), still ranked as the third most predictive feature in the adult SM model. The presence of several DCs in the top ranks of our models is remarkable. This is because RF analysis may penalize DC domains, as the algorithm recognizes them as a proxy of each other, decreasing the MDCA score. The importance of DC8 (EPCR), DC6 (C-terminal), and DC19 (CD36) in both the adult and children SM models suggest that these domains play an important role in severe disease across age groups.

10.1128/mBio.00217-19.5 TABLE S3 MDCA and mProbes FWER for each *var* domain subtype in adult and childhood UM versus SM models. Download Table S3, PDF file, 0.1 MB. Copyright © 2019 Duffy et al. 2019 Duffy et al. This content is distributed under the terms of the Creative Commons Attribution 4.0 International license.

Receiver operating characteristic (ROC) curves and the accompanying 95% confidence intervals (CIs) representing the unbiased out-of-bag (OOB) predictions made during model training are shown in Fig. 3C. All *var* profile models significantly discriminate UM from SM. Notably, the combined childhood SM model (area under the ROC curve [AUC], 0.75 [95% CI, 0.68 to 0.82]) performs very similarly to the individual Tanzanian and Malawian models (TZ AUC, 0.81 [95% CI, 0.73 to 0.9]; BLZ AUC, 0.75 [95% CI, 0.65 to 0.84]) and the adult Goan model (AUC of 0.72 [95% CI, 0.62 to 0.83]). Taken together, predictive performance was highly consistent across all sites.

To further explore pathogenic mechanisms in children and adults, we made blind predictions, using the adult model to classify the child samples and the childhood model to classify the adult samples. Importantly, both models showed significant ability to cross-classify (Fig. 3D) for both children on adults (AUC, 0.66 [95% CI, 0.55 to 0.78]; *P* = 0.002) and adults on children (AUC, 0.69 [95% CI, 0.62 to 0.78]; *P* = 5.3 × 10^−7^). In other words, adult SM could be predicted by *var* profiling of pediatric samples and vice versa. This demonstrates that, despite presenting highly distinct severe disease manifestations, similar *var* subsets are predictive of SM in both adults and children.

### Parasite biomass and *var* profiles are independent and complementary contributors to severe disease in both adults and children.

A recurrent question in the malaria pathogenesis field is the relative contribution of parasite biomass and parasite cytoadhesion to malaria severity. PfHRP2 measurements were available for both the Malawi and Goa cohorts, but not Tanzania. PfHRP2 levels alone significantly discriminated severe from uncomplicated malaria in both Malawi (AUC, 0.85 [95% CI, 0.79 to 0.92]; *P* = 4 × 10^−9^) and Goa (AUC, 0.7 [95% CI, 0.59 to 0.81]; *P* = 0.0018) (Fig. 3E). To evaluate the improvement resulting from the combination of PfHRP2 and the *var* gene models, logistic regression models of the form severity = PfHRP2 + *var* signature score were fit. Receiver operating characteristic (ROC) curves for these models are shown in Fig. 3E, and the combined severity predictions significantly improved on both PfHRP2 alone and *var* profiles alone for both Malawi (χ^2^ test, *P* = 0.0005) and Goa (χ^2^ test, *P* = 0.006). Statistical analyses indicate substantial improvement in all prediction metrics in both sites when *var* profile models are combined with PfHRP2 measurements (Malawi, *var* profile alone odds ratio [OR], 6.6, HRP2 alone OR, 15.9, and *var* profile plus HRP2 OR, 28.3; Goa, *var* profile alone OR, 5.77, HRP2 alone OR, 3.75, and *var* profile plus HRP2 OR, 15) (see Table S4 in the supplemental material). This demonstrates that *var* profiles and parasite load are independent and complementary biomarkers of malaria severity in two independent study cohorts.

10.1128/mBio.00217-19.6 TABLE S4 Summary statistics for combining *var* profile models with PfHRP2 measurements for Malawi and Goa samples. Download Table S4, PDF file, 0.1 MB. Copyright © 2019 Duffy et al. 2019 Duffy et al. This content is distributed under the terms of the Creative Commons Attribution 4.0 International license.

### Parasite load is the primary factor underlying adult SM cases with multiple clinical complications.

**FIG 4:**
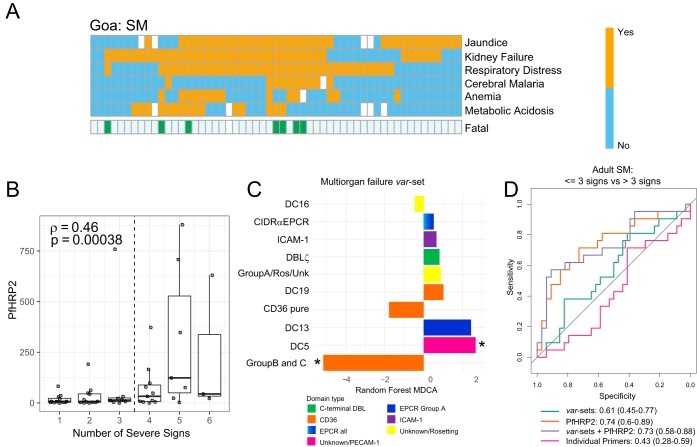
Plasma PfHRP2 levels are associated with the number of severe malaria criteria in adults. (A) Heat map showing prevalence of clinical signs of adult SM in Goa. Fatal SM cases are indicated below. A white box indicates missing information. (B) Box and dot plot of plasma PfHRP2 levels in adult SM, stratified by number of severity criteria. The median is indicated by a horizontal line. (C) Bar plot showing importance of primer sets in the severe criterion count *var* set model. Negative MDCAs indicate sets with lower expression in patients with >3 severity criteria. (D) ROC curves showing the predictive power of individual *var* domain subtypes, PfHRP2 levels, *var* domain subtypes grouped by binding phenotype (*var* sets), and *var* sets combined with PfHRP2 levels to classify adult SM patients as having up to three clinical signs versus those with over three clinical severe signs.

Adult SM is frequently associated with multiorgan complications that increase fatality risk (2). Within our Goan cohort, acute respiratory distress syndrome, acute kidney injury, and jaundice were the most frequent complications and were frequently found together (Fig. 4A). Notably, the number of severe malaria criteria is significantly correlated with PfHRP2 levels (Spearman’s ρ = 0.46; *P* = 0.00038 [Fig. 4B]), presenting a pronounced increase in individuals with more than three severity criteria. Fatal SM cases were also associated with higher burdens of severe criteria, with six of seven fatal cases in this cohort presenting with more than three severity criteria (Fig. 4A).

**FIG 5:**
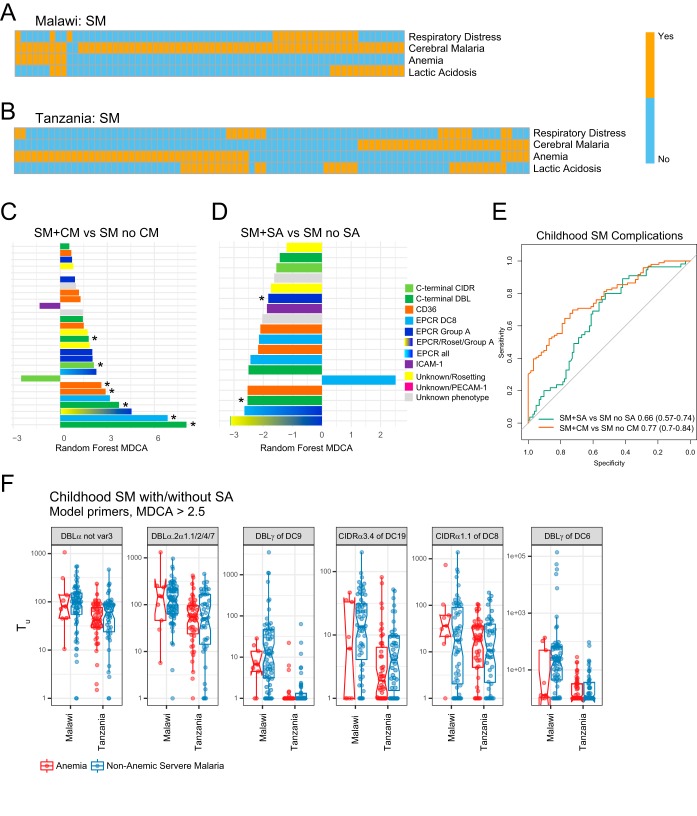
*var* transcript profiles distinguish childhood coma (CM) and anemia (SA). (A and B) Heat maps showing the prevalence of clinical signs of childhood SM in Malawi and Tanzania, respectively. Patients without a WHO criteria listed on the heat map presented hyperparasitemia. (C and D) Bar plots showing *var* domain subtype importance (MDCA, negative bars indicate lower primer expression in SM plus CM or SM plus SA patients) for the SM plus CM versus SM with no CM and the SM plus SA versus SM with no SA models. (E) ROC curves of RF models discriminating CM and SA from other SM patients in the combined Malawi and Tanzania sets. (F) Box plots of site-specific expression for the most important (MDCA, >2.5) *var* domain subtypes in the anemia model.

An RF model was trained to classify adult severe malaria cases into those with up to three severity criteria and those with over three severity criteria. Notably, the model based only on *var* profile data showed no discriminatory power (AUC, 0.43 [95% CI, 0.28 to 0.59]), while PfHRP2 levels alone successfully discriminated patients with over three severity criteria (AUC, 0.74 [95% CI, 0.58 to 0.88]) (Fig. 4). As the redundant nature of the primer repertoire used in this study could artificially decrease the importance of some *var* domains contributing to SM by acting as proxies for each other during modeling, we also trained a model based on primers grouped into related *var* predicted adhesion types (Fig. 5D; see Table S5 in the supplemental material). T_u_ values of *var* sets were summed to construct a summarized matrix of discrete, nonredundant binding phenotype sets. Patients with multiorgan complications presented decreased expression of *var* genes with a predicted CD36 binding phenotype and increased expression of a subset of group A *var* genes (DC13 EPCR binders and DC5-containing genes, which bind PECAM-1 in some instances). Conversely, the DC8 EPCR-binding subset did not predict multiorgan complications, as their expression was elevated among all adult SM patients (Fig. 4C). While the *var* set model showed improved predictive power (AUC, 0.61 [95% CI, 0.45 to 0.77]), it did not reach the threshold for statistical significance. Moreover, there was no improvement over PfHRP2 alone (Fig. 4D). Taken together, this analysis suggests that P. falciparum growth and expansion are the primary parasite factors that influenced presentation of multiple severity signs in adults.

10.1128/mBio.00217-19.7 TABLE S5 Classification of targeted domains into summed *var* sets corresponding to specific domain cassettes, binding phenotypes, and domain positions. Download Table S5, PDF file, 0.1 MB. Copyright © 2019 Duffy et al. 2019 Duffy et al. This content is distributed under the terms of the Creative Commons Attribution 4.0 International license.

### Specific *var* profiles are associated with childhood severe malaria and anemia.

To investigate *var* profile associations with childhood disease complications, we analyzed SM samples from the combined Tanzanian and Malawi data sets. The main SM complications in children are coma (cerebral malaria [CM]), severe anemia (SA), lactic acidosis, and respiratory distress, which can appear alone or in combination (Fig. 5A and B). While the Tanzanian cohort was selected for an even distribution of complications, the Malawian cohort is biased toward CM patients, as it was the main focus of the original study (34). Fitting an RF model to the combined cohorts accurately classified CM from non-CM among SM cases (AUC, 0.77 [95% CI, 0.7 to 0.84] [Fig. 5C to E]). Consistent with previous studies (33, 34, 38), childhood CM is associated with higher levels of transcripts encoding group A and DC8 EPCR binders (group A: DBLα not *var3*; DC8: DBLγ4/6, DBLβ12 and DBLβ3/5, and CIDRα1.1; and DC8-group A: DBLα2/α1.1/2/4/7) (Fig. 5C; see Table S6 in the supplemental material). We also observed higher levels of transcripts encoding C-terminal domains (DBLγ of DC6 and DC9), as well as the DC19 subset of CD36-binding domains (Fig. 5C; Table S6).

10.1128/mBio.00217-19.8 TABLE S6 MDCA and mProbes FWER for each *var* domain subtype in childhood coma (cerebral malaria [CM]) and anemia (SA) models. Download Table S6, PDF file, 0.1 MB. Copyright © 2019 Duffy et al. 2019 Duffy et al. This content is distributed under the terms of the Creative Commons Attribution 4.0 International license.

This modeling approach also significantly discriminated SA from non-SA among SM patients (AUC, 0.66 [95% CI, 0.57 to 0.74] [Fig. 5D and E]), with SA being associated with lower expression of most adhesion domains present in the CM model, including group A EPCR binders, the DC6 and DC9 C-terminal domains, and the DC19 subset of CD36 binders (Fig. 5D; Table S6). To further investigate if the performance of the SA model was simply being driven by lower *var* transcription levels in parasites associated with SA cases or study site-specific biases, we compared transcript levels of the most informative *var* domain subtypes (MDCA, >2.5) from the SA model (Fig. 5F). This analysis indicated that while most *var* domains were expressed at lower levels in SA, the DC8 domain, CIDRα1.1 (Table S6), had higher median transcript levels in SA than CM cases in both Malawi and Tanzania. However, CIDRα1.1 DC8 transcripts were also elevated in many CM cases, suggesting that DC8-expressing EPCR-binding parasites are linked to both CM and SA.

## DISCUSSION

The *var* gene family plays a central role in parasite immune evasion and pathogenesis and has extensively diversified under immune pressure. The motivation of this study was to evaluate if the same *var* subsets were linked to severe malaria in Africa and India and to explore how *var* adhesion types interact with parasite biomass to influence malaria disease presentation in children and adults. This work represents the most comprehensive analysis to date of parasite factors leading to SM. This has been accomplished by assembling a multisite adult and pediatric data set of *var* profiles, comprising hundreds of patient samples. The *var* profiles studied in this meta-analysis were tested with a panel of domain-specific primers that provide broad coverage of the *var* repertoire (estimated 87% of *var* genes) (33). While these primers have less overlapping domain coverage of the CD36-binding PfEMP1 subset, which comprise 90% of the P. falciparum *var* genomic repertoire, independent next-generation sequencing of *var* amplicons of the pediatric cohorts analyzed in this study (34, 38) validated the use of the *var* domain-specific primers to determine patient *var* profiles.

Our central observation is that both parasite biomass and *var* profiles are important, independent, and complementary in the development of adult and pediatric SM. Furthermore, despite *var* transcript profiles showing extensive within-host and between-site differences, the *var* profile signatures associated with severe malaria in African children are highly predictive of severe malaria in Indian adults and vice versa. Most previous *var* transcript profiling has focused on African children, and less investigation has been conducted in lower-transmission settings where disease affects both children and adults. Despite differences in disease presentation between children and adults, severe malaria in both Indian adults and African children is clearly linked to increased transcription of variants predicted to bind EPCR, including DC8 and group A *var* genes. This demonstrates the power of this meta-analytical approach to reveal a universal signature of severe malaria and validates the results of previous studies implicating the DC8 and group A *var* subsets in severe malaria (33–38).

In contrast to the clear associations of specific *var* transcript profiles with severe malaria, the relationship of specific binding phenotypes and malaria disease syndromes is more complex. Understanding this relationship is complicated by the complex presentation of severe malaria in children and adults, as many patients had more than one severity criterion, especially in our adult Indian cohort. The most definitive link to *var* adhesion types was observed for childhood malaria, in which children with coma and severe anemia had distinctive *var* signatures. While predicted EPCR-binding group A *var* transcripts were lowest in UM cases, intermediate in SA, and highest in CM, DC8 *var* transcript levels tended to be higher in SA than CM cases, indicating there may be subtle differences in the transcript level or composition of DC8 variants between the two syndromes. However, the incidence of anemia in this study is highly skewed: it is common in our Tanzanian cohort and uncommon in the Malawian cohort. Along with this, the most anemic individuals also were not cerebral malaria patients. This raises the possibility of site-specific and CM expression patterns biasing the signature. Nevertheless, the specific association of DC8 *var* expression with severe anemia, and of DC8 and group A *var* with CM, is intriguing. DC8 PfEMP1 binds EPCR and most likely a range of other not yet well understood human receptors (46), while some group A *var* genes encode dual adhesive properties for EPCR and ICAM-1 (21, 23), as well as other yet undefined human receptors. This is suggestive of a mechanistic hypothesis in which malaria coma is potentially driven by both ICAM-1 and EPCR binding parasites, in contrast with anemia. Further study is needed to disentangle these effects.

In addition to *var* disease signatures, our study confirms the strong role of parasite biomass in severe disease (39–43). RF models reveal that both factors are independent and complementary biomarkers of severity. The combination of both features increases the sensitivity, specificity, and odds ratio of severe malaria prediction in both pediatric and adult severe malaria. An expansion of the circulating and sequestering parasite population might directly contribute to disease severity by directly increasing endothelial cell activation (47–49) and microvascular obstruction (4, 50).

Whereas EPCR-binding *var* subsets were linked to severe malaria in adults, progression to multisymptomatic disease (greater than three severity criteria) appeared to be primarily driven by parasite load. However, we did see a possible reduction in CD36 binders in adults with multiple severe malaria complications. This may suggest that an overall increase in EPCR-binding parasites contributes to multiorgan complications in adult SM. Future work is needed to address whether specific *var* adhesion types are linked to the distinct organ complications of adult SM.

A limitation of this study is that the meta-analysis focuses on P. falciparum transcription differences in severe malaria and lacks experimental assessment of parasite binding. Nevertheless, the cytoadhesion predictions for CD36, EPCR, and ICAM-1 are supported by multiple *in vitro* studies of both laboratory-adapted P. falciparum lines (21–23, 26–28) and cerebral malaria isolates (16, 51, 52). The consistency in the importance of group A and DC8 *var* transcripts as a predictor of disease severity in patients of multiple ages and ethnicities, presenting multiple disease syndromes, suggests an important role of EPCR in severe disease. This likely reveals a pathogenic pathway, as IE binding to EPCR is proposed to prevent its vascular homeostatic and protective function (53).

In summary, machine learning meta-analysis has revealed common *var* signatures of severe malaria in African children and Indian adults and further suggests important interactions between parasite biomass and *var* adhesion type in disease presentation.

## MATERIALS AND METHODS

### Ethical approval.

Informed consent was obtained from all study participants. The Indian study was approved by the ethics boards at Goa Medical College and Hospital, the University of Washington, and the Western Institutional Review Board, used on behalf of the Center for Infectious Disease Research/Seattle Children’s Research Institute, as well as by the Government of India Health Ministry Screening Committee. The Malawian study (34) was approved by the institutional review boards at the University of Malawi College of Medicine, Michigan State University, and the Albert Einstein College of Medicine. The Tanzania study (33) was approved by the Tanzania Medical Research Coordinating Committee, and parents or guardians of children provided consent.

### Composition of human cohorts and patient recruitment.

Newly recruited patients were obtained from the Goa study site, using the previously published sample collection protocol (37). Additional subjects were recruited between August 2012 and January 2017 from the hospital admissions or outpatient wards at the Goa Medical College and Hospital. P. falciparum infections were identified using Giemsa-stained thick and thin smears for parasitemia determination and species identification. Plasmodium vivax infections were excluded from the study. After informed consent was obtained, blood samples from P. falciparum-infected patients were collected in acid citrate dextrose vacutainers and separated into plasma and red blood cell fractions in RNAlater, before freezing at −80°C. Adult SM was defined as hospitalization with any of the following signs: coma (Glasgow coma score, <10), severe anemia (Hb, <7 g/dl), jaundice (bilirubin, >3 mg/dl), kidney failure (serum creatinine of >3 mg/dl or blood urea nitrogen of >17 mmol/liter), shock (systolic blood pressure of <80 mm Hg plus cold extremities), metabolic acidosis (peripheral venous bicarbonate, <155 mmol/liter), respiratory distress (>20 breaths per min or partial pressure of oxygen in alveoli [PaO_2_] of <75 mm Hg), or hypoglycemia (blood glucose, <40 mg/dl). Unadmitted patients were classified as UM. For the two African children sites, SM was defined as hospitalization with any of the following presentations: coma (Blantyre coma score, ≤2), severe anemia (Hb, <5 g/dl), and lactic acidosis (plasma lactate, >5 mmol/liter) as published previously (33, 34). Pediatric patients with respiratory distress were classified if any of the following variables was present: deep breathing, grunting, nasal flaring, chest indrawing, and chest retractions (for BLZ) or Kussmaul breathing and being X-ray positive (for TZ).

### qRT-PCR measurement of *var* transcription profiles.

All participants had quantitative reverse transcription-PCR (qRT-PCR)-based *var* transcript profiling performed as previously described (33). Briefly, TRIzol reagent was used to extract RNA from parasitized red blood cells, and cDNA was synthesized. The *var* primer threshold cycle (*C_T_*) values were quantified by qRT-PCR using a panel of degenerate primers designed to identify known *var* domain subclasses. Levels of *var* expression were normalized relative to the housekeeping gene primers adenylosuccinate lyase and seryl-tRNA synthetase (GMC) and aldolase and seryl-tRNA synthetase (TZ and BLZ): Δ*C_T var_*_primer_ = *C_T var_*_primer_ − mean *C_T_*_housekeeping primers_. Low-abundance transcripts with a Δ*C_T_* of >5 were set to 5. Samples where housekeeping primers showed a mean *C_T_* of ≥ 25 (TZ) or >30 (BLZ and GMC) were excluded from further analysis. For all subsequent analyses, *var* expression was represented as transcript units [T_u_ = 2^(5 − Δ^*^CT^*^)^], where a T_u_ value of 1 represents a 0 level of expression.

### PfHRP2 plasma quantification.

PfHRP2 quantification was performed as previously described (34, 37). Briefly, BLZ samples were analyzed by a commercial kit (Cellabs, Brookvale, Australia) and for the Goan samples (GMC), a double-site sandwich enzyme-linked immunosorbent assay (ELISA) was performed. The standard curve for Goan samples was established using purified PfHRP2 protein (kindly donated by David Sullivan, Johns Hopkins Bloomberg School of Public Health).

### Statistical and machine learning analysis.

All statistical and machine learning analysis was carried out using the R language for statistical computing (54). All primer expression and distribution plots were created using the R ggplot2 library (55).

**(i) Clustering and distribution analysis.** Primer-primer correlation coefficients were calculated as Spearman’s rho (ρ), and hierarchical clustering was performed using the R hclust function. Significant differences in primer distribution between sites were evaluated using the Kolmogorov-Smirnov test, using the R ks.test function, and *P* values were adjusted for multiple testing using the Holm approach.

**(ii) Machine learning modeling and significance testing.** Machine learning models were used to assess the ability of *var* transcript profiles to classify subjects as SM or UM or to distinguish specific SM severity criteria. RF models were trained to distinguish SM from UM in all three sites using the R randomForest package (56). Missing primer T_u_ values were set to be equal to 1 (indicating primer expression below the limit of detection). To avoid bias due to overfitting, unbiased model predictive performance was evaluated based on the consensus RF out-of-bag (OOB) predictions generated during model training. Receiver operating characteristic (ROC) curves were constructed based on the OOB predictions. Strength of predictive performance was measured as area under the ROC curve (AUC). This takes the form of a number between 0 and 1, where 0.5 represents random performance and 1 represents perfect classification. The R pROC (57) package was used to estimate 95% confidence intervals (CIs) around the AUC. Models were considered significant when the lower 95% CI was over 0.5 (i.e., the entire 95% confidence interval of the AUC was in the “better than random” area of the ROC curve). *P* values for ROC curves were calculated by fitting two logistic regression models—one of the form class ∼ signature_score and the other of the form class ∼1—and comparing them with the R anova.glm(test=“Chisq”) function. Sensitivities, specificities, and odds ratios were calculated from confusion matrices of binary predictions made at the optimal point on the ROC curve, defined as the point that minimized the Euclidean distance to a sensitivity and specificity of 1.

To estimate the contribution of individual *var* subtypes to model performance, the mean decrease in classifier accuracy (MDCA) was used. This statistic is calculated during the resampling training procedure of the RF model and is the percentage of decrease in accuracy in random data subsamples that contain the *var* subtype versus those that do not. Significance of model *var* domain subtypes was calculated using the mProbes algorithm (58), which compares model subtypes to randomly shuffled subtypes to calculate a family-wise error rate (FWER).

### Creation of summarized *var* primer sets.

Sets of *var* domain subtypes (*var* sets) that combine related information, such as a common binding phenotype (e.g., EPCR or CD36), gene group (e.g., group A, B, or C), domain organization (C-terminal DBL or C-terminal CIDR), or part of a tandemly arrayed domain combination (e.g., DC8/DC13) were developed (Table S5) and applied to summarize the *var* transcript levels. The summed *var* transcript abundance (*var* set) was calculated by summing the reported T_u_ values for each *var* domain subtype per sample and subtracting 1 for each additional primer beyond the first. This was done to ensure that the set’s summarized T_u_ value was equal to 1 if no expression was observed for any *var* domain subtypes in a particular sample. Machine learning and importance estimates were created for the summarized features, as described above.
