# Supplementary material for: Meta-analysis of Plasmodium falciparum var Signatures Contributing to Severe Malaria in African Children and Indian Adults
Source: mBio. 2019 Apr 30;10(2):e00217-19. doi: 10.1128/mBio.00217-19 (PMC6495371; doi:10.1128/mBio.00217-19)
Supplement: TABLE S1 [file mBio.00217-19-st001.pdf]

Table S1: Demographic and clinical characteristics of the *var*-profiling cohorts

	India (GMC)			Malawi (BLZ)			Tanzania (TZ)		
	**SM** (n=55)	**UM** (n=37)	*p* *	**SM** (n=68)	**UM** (n=40)	*p* *	**SM** (n=90)	**UM** (n=32)	*p* *
Age (median, IQR)	27 (22-36.5)	25 (20 -38)	0.22	4 (2-6)	4.5 (2-7)	0.26	2 (1-3)	2 (1-3)	0.63
Male %	89%	89%	0.99	60%	58%	0.97	57%	53%	0.73
Parasite density (median, IQR)	19,091 (5,645-61,740)	12,644 (7,022-38,292)	0.66	29.160 (741-109,920)	-	-	24,518 (2,878 – 138,565)	43,193 (16,721-69,825)	0.47
Hb (median, IQR)	10.2 (7.5 – 11.4)	11.8 (10.1 - 13.4)	**$8.3x10^{-4}$**	7.7 (6.6 - 9.2)	9.7 (8.2-11.5)	**$2.14x10^{-4}$**	4.8 (3.9-6.9)	7.85 (6.38 – 8.38)	**$8.6x10^{-7}$**
Lactate (median, IQR)	-	-	-	3.3 (2.1 – 4.7)	-	-	3.3 (2.77 – 4.1)	4.4 (3.23 – 5.62)	**$8.5x10^{-4}$**
PfHRP2 (median, IQR)	15.6 (4.22 – 55.8)	3.75 (4.69-11.9)	**1.9×10^{-3}**	3,200 (540 – 10,254)	190 (46.2 - 454)	**$1.4x10^{-9}$**	-	-	-
Glasgow coma score (median)	14	15	**2.0×10^{-7}**	-	-	-	-	-	-
Blantyre coma score (median)	-	-	-	2	5	**$5.8x10^{-18}$**	5	5	**$5.4x10^{-6}$**
Number of complications (mean)	2.7	-	-	2	-	-	1.4	-	-
Mortality %	13%	0%	**0.024**	10%	0%	0.12	10%	0%	0.065

* UM vs SM, Wilcoxon p-value
