# Supplementary material for: Meta-analysis of Plasmodium falciparum var Signatures Contributing to Severe Malaria in African Children and Indian Adults
Source: mBio. 2019 Apr 30;10(2):e00217-19. doi: 10.1128/mBio.00217-19 (PMC6495371; doi:10.1128/mBio.00217-19)
Supplement: TABLE S2 [file mBio.00217-19-st002.pdf]

Table S2. Broad phenotypic classification of domains targeted by individual *var* primers and associated color scheme

Category	DC	Current name	Color
**CD36**		**CIDRα.3.1-3**	
**CD36**		**CIDRα2.2**	
**CD36**		**CIDRα2.3/5/6/7/9/10**	
**CD36**	19	**CIDRα3.4 of DC19**	
**CD36**		**DBLα.0.1**	
**CD36**	19	**DBLα0.16 of DC19**	
**CD36**		**DBLα0.6/9**	
**CD36**	20	**DBLα0.9 of DC20**	
**C-terminal CIDR**		**CIDRγ**	
**C-terminal CIDR**		**CIDRγ1/2**	
**C-terminal CIDR**		**CIDRγ2.9**	
**C-terminal DBL**	12	**DBLε12 of DC12**	
**C-terminal DBL**	7	**DBLε2 of DC7**	
**C-terminal DBL**	7	**DBLε3 of DC7**	
**C-terminal DBL**	6	**DBLζ5 of DC6**	
**C-terminal DBL**	6	**DBLζ5.3 of DC6**	
**C-terminal DBL**	9	**DBLγ of DC9**	
**C-terminal DBL**	9	**DBLγ9**	
**C-terminal DBL**	9	**DBLζ4 of DC9**	
**C-terminal DBL**	10	**DBLζ6 of DC10**	
**EPCR all**		**CIDRα1.4 and CIDRα1**	
**EPCR all**		**DBLα2/α1.1/2/4/7**	
**EPCR DC8**	8	**CIDRα1.1 of DC8**	
**EPCR DC8**	8	**DBLα-CIDRα of DC8**	
**EPCR DC8**	8	**DBLβ12 and DBLβ3.5**	
**EPCR DC8**	8	**DBLγ4/6 of DC8**	
**EPCR Group A**	13	**CIDRα1.4 of DC13**	
**EPCR Group A**		**CIDRα1.6**	
**EPCR Group A**		**CIDRα1.7**	
**EPCR Group A**		**DBLα1.4**	
**EPCR Group A**	13	**DBLα1.7 of DC13**	
**Group A Rosetting/ EPCR/ PECAM-1/ Unknown**		**DBLα not var3**	
**ICAM-1**		**DBLβ3**	
**ICAM-1**		**DBLβ5**	
**Unknown / Rosetting**	16	**CIDRδ of DC16**	
**Unknown / Rosetting**	16	**DBLα 1.5/6b of DC16**	
**Unknown / Rosetting**	16	**DBLα1.5/6a of DC16**	
**Unknown / PECAM-1**	5	**DBLβ7 & 9 of DC5**	
**Unknown / PECAM-1**	5	**DBLγ of DC5**	
**Unknown phenotype**	1	**DBLα1.1 of DC1**	
**Unknown phenotype**	3	**DBLε8 of DC3**	
