# Supplementary material for: Meta-analysis of Plasmodium falciparum var Signatures Contributing to Severe Malaria in African Children and Indian Adults
Source: mBio. 2019 Apr 30;10(2):e00217-19. doi: 10.1128/mBio.00217-19 (PMC6495371; doi:10.1128/mBio.00217-19)
Supplement: TABLE S3 [file mBio.00217-19-st003.pdf]

Table S3. MDCA and mProbes FWER for each *var* domain subtype in adult and childhood UM versus SM models.

			Adults (Goa) SM vs UM		Children (TZ + BLZ) SM vs UM	
**Primer**[a]	**Group**	**Category**	mProbes FWER	Signed MDCA	FWER	Signed MDCA
**DBLα not var3 (Group A)**	A		0.96	1.46	0	13.39
**DBLα2/α1.1/2/4/7**	B/A & A		0.83	1.53	0.03	7.84
**DBLβ12 and DBLβ3.5**	B.A (DC8)		0.34	1.84	0.03	6.50
**DBLα-CIDRα of DC8**	B/A (DC8)		0.1	1.32	0.09	4.65
**CIDRα1.1 of DC8**	B/A (DC8)		0.38	1.50	0.33	4.08
**DBLα1.1 of DC1**	A		1	0.95	0.47	3.73
**DBLζ4 of DC9**	B		1	1.04	0.64	-3.47
**CIDRγ**	B		0.71	-1.63	0.99	2.77
**DBLβ3**	A		0.87	0.99	0.65	2.73
**DBLγ of DC9**	A,B,C		1	-0.87	0.86	-2.49
**CIDRα2.3/5/6/7/9/10**	B		1	-1.20	0.97	2.20
**CIDRα3.4 of DC19**	B,C		1	1.31	0.99	2.18
**CIDRγ1/2**	B		0.95	-1.28	0.99	-2.18
**DBLγ4/6 of DC8**	B/A (DC8)		0.06	2.16	0.99	2.02
**DBLβ5**	B (A,C)		0.04	1.02	0.75	2.00
**DBLα0.6/9**	B		1	-1.16	0.96	1.95
**DBLα1.5/6a of DC16**	A		1	0.95	1	1.91
**DBLα1.7 of DC13**	A		0.72	1.12	0.93	1.87
**CIDRα2.2**	B		0.99	-1.06	0.94	1.86
**DBLα0.16 of DC19**	B		0.39	1.38	1	1.83
**DBLγ of DC6**	B (A/C)		0.25	1.79	1	1.73
**CIDRα1.4 and CIDRα1**	A		0.8	1.06	0.97	1.66
**DBLα 1.5/6b of DC16**	A		1	0.77	0.99	1.65
**DBLζ5 of DC6**	B (A, C)		1	0.93	1	1.65
**DBLα0.9 of DC20**	B		1	0.93	0.99	1.64
**CIDRα1.4 of DC13**	A		0.99	0.78	0.34	1.64
**CIDRα.3.1-3**	B,C		1	1.15	0.99	1.59
**CIDRα1.7**	A		1	0.70	0.96	1.54
**CIDRδ of DC16**	A		1	0.85	0.8	1.43
**DBLγ of DC5**	A		0.97	0.00	0.97	1.39
**DBLε8 of DC3**	A		1	0.93	0.79	1.28
**DBLε2 of DC7**	B (C)		0.98	-1.08	0.83	1.02
**DBLε3 of DC7**	B (C)		0.94	0.88	1	0.94
**DBLα.0.1**	B		0.81	0.69	1	0.57
**DBLβ7 & 9 of DC5**	A		0.87	-0.81	1	0.56
**DBLε12 of DC12**	B,A		1	-0.77	1	0.00
**DBLγ9**	A,B,C		1	0.00	1	0.00

[a] Primers are ordered from highest to lowest mean decrease in classifier (MDCA) values in the children comparison. Postive and negative indicates the direction of transcriptional differences. FWER = family wise error rate.
