# Supplementary material for: Meta-analysis of Plasmodium falciparum var Signatures Contributing to Severe Malaria in African Children and Indian Adults
Source: mBio. 2019 Apr 30;10(2):e00217-19. doi: 10.1128/mBio.00217-19 (PMC6495371; doi:10.1128/mBio.00217-19)
Supplement: TABLE S4 [file mBio.00217-19-st004.pdf]

Table S4: Summary statistics for combining var-profile models with PfHRP2 measurements for Malawi and Goa samples

	p (χ^2)	Odds ratio	Sensitivity	Specificity
**Malawi *var*-profile model**	1.78E-05	6.61	0.76	0.68
**Malawi PfHRP2**	4.84E-09	15.87	0.79	0.82
**Malawi PfHRP2+*var*-profile model**	5.45E-11	28.3	0.82	0.87
**Goa *var*-profile model**	0.00030484	5.77	0.65	0.76
**Goa PfHRP2**	0.00175578	3.75	0.74	0.57
**Goa PfHRP2+*var*-profile model**	4.09E-06	15	0.8	0.8
