# Supplementary material for: Meta-analysis of Plasmodium falciparum var Signatures Contributing to Severe Malaria in African Children and Indian Adults
Source: mBio. 2019 Apr 30;10(2):e00217-19. doi: 10.1128/mBio.00217-19 (PMC6495371; doi:10.1128/mBio.00217-19)
Supplement: TABLE S5 [file mBio.00217-19-st005.pdf]

Table S5. Classification of targeted domains into summed *var* sets corresponding to specific doamin cassettes, binding phenotypes and domain positions.

var -set	Category 1 (Main)	Category 2	Category 3	Category 4	Color
CD36 pure	**CD36**				
DC19	**CD36**				
DC20	**CD36**				
Group B and C	**CD36**	**C-terminal DBL**	**C-terminal CIDR**		
C-terminal CIDR	**C-terminal CIDR**				
C-terminal DBL	**C-terminal DBL**				
DBLε	**C-terminal DBL**				
DBLζ	**C-terminal DBL**				
DC10	**C-terminal DBL**				
DC12	**C-terminal DBL**				
DC6	**C-terminal DBL**				
DC7	**C-terminal DBL**				
DC9	**C-terminal DBL**				
IgM pure	**C-terminal DBL**	**C-terminal CIDR**			
CIDRα EPCR	**EPCR all**				
DC8	**EPCR DC8**				
DC A	**EPCR**	**Rosetting**	**PECAM-1**	**Unknown phenotype**	
DC13	**EPCR**				
ICAM1	**ICAM-1**				
DC16	**Unknown**	**Rosetting**			
DC5	**Unknown**	**PECAM-1**			
GroupA Ros-Unk	**Unknown**	**Rosetting**			
DC1	**Unknown phenotype**				
DC3	**Unknown phenotype**				
Unknown.pseudo	**Unknown phenotype**				
