# Supplementary material for: Meta-analysis of Plasmodium falciparum var Signatures Contributing to Severe Malaria in African Children and Indian Adults
Source: mBio. 2019 Apr 30;10(2):e00217-19. doi: 10.1128/mBio.00217-19 (PMC6495371; doi:10.1128/mBio.00217-19)
Supplement: TABLE S6 [file mBio.00217-19-st006.pdf]

Table S6. MDCA and mProbes FWER for each *var* domain subtype in childood coma (CM) and anemia (SA) models.

			Severe Anemia (SA)		Cerebral Malaria (CM)	
**Primer**[a]	**Group**	**Category**	**mProbes FWER**	**Signed MDCA**	**mProbes FWER**	**Signed MDCA**
**DBLγ of DC9**	A,B,C		0.08	-2.54	0.01	8.04
**DBLγ4/6 of DC8**	B/A (DC8)		0.77	-2.14	0.00	6.85
**DBLα not var3 (Group A)**	A		0.88	-3.13	0.39	4.54
**DBLγ of DC6**	B (A,C)		0.33	-2.50	0.03	3.74
**DBLβ12 and DBLβ3.5**	B/A (DC8)		0.89	-2.44	0.98	3.17
**CIDRα3.4 of DC19**	B,C		0.53	-2.54	0.16	2.89
**DBLα0.16 of DC19**	B		0.92	-2.10	0.24	2.62
**DBLα2/α1.1/2/4/7**	B/A & A		0.90	-2.64	0.91	2.31
**DBLα0.6/9**	B		1.00	-1.87	0.98	2.15
**CIDRγ1/2**	B		0.81	-1.55	0.14	2.14
**DBLα1.7 of DC13**	A		1.00	-1.51	0.38	2.06
**CIDRα1.6**	A		0.11	-1.83	0.26	2.04
**DBLγ of DC5**	A		1.00	-1.20	0.59	1.88
**CIDRα1.1 of DC8**	B/A (DC8)		0.98	2.51	1.00	1.82
**DBLε2 of DC7**	B (C)		0.72	-1.44	0.07	1.82
**DBLα1.5/6a of DC16**	A		1.00	-1.74	0.43	1.75
**CIDRα2.3/5/6/7/9/10**	B		0.99	-2.17	0.96	1.50
**DBLζ4 of DC9**	B		1.00	-1.46	0.58	1.44
**DBLα1.1 of DC1**	A		0.89	-2.03	0.96	1.44
**DBLβ5**	B (A,C)		1.00	1.36	0.98	-1.31
**CIDRα.3.1-3**	B,C		1.00	-1.69	0.74	1.30
**DBLα0.9 of DC20**	B		1.00	-1.49	0.81	1.19
**DBLβ3**	A		0.99	-1.87	1.00	1.19
**DBLζ5 of DC6**	B (A,C)		1.00	-1.51	0.98	1.18
**DBLε8 of DC3**	A		0.73	-1.63	0.70	1.00
**CIDRα1.7**	A		1.00	-1.30	0.96	0.94
**CIDRα1.4 and CIDRα1**	A		0.99	-1.57	1.00	0.91
**CIDRδ of DC16**	A		1.00	-1.61	1.00	0.87
**DBLα 1.5/6b of DC16**	A		1.00	-1.66	0.90	0.83
**CIDRα1.4 of DC13**	A		1.00	-1.17	0.95	0.75
**CIDRα2.2**	B		1.00	-1.20	0.95	0.71
**DBLζ6 of DC10**	B		1.00	-1.08	0.99	0.61
**DBLε3 of DC7**	B (C)		1.00	-1.01	0.99	0.58
**DBLε12 of DC12**	B,A		0.99	0.98	1.00	-0.55
**DBLβ7 & 9 of DC5**	A		1.00	-0.84	1.00	0.32
**DBLα.0.1**	B		0.99	-0.87	1.00	0.31

[a] Primers are ordered from highest to lowest mean decrease in classifier (MDCA) values in the cerebral malaria comparison. Positive and negative indicates the direction of transcriptional differences. FWER = family wise error rate.
